# Translating Alcohol Research: Opportunities and Challenges

**DOI:** 10.35946/arcr.v37.1.01

**Published:** 2015

**Authors:** Angela M. Batman, Michael F. Miles

**Affiliations:** Angela M. Batman, Ph.D., is a postdoctoral researcher in the Department of Pharmacology/Toxicology at Virginia Commonwealth University (VCU), Richmond, Virginia, and director of behavioral pharmacology at Melior Discovery, Exton, Pennsylvania.; Michael F. Miles, M.D., Ph.D., is a professor in the Departments of Pharmacology/Toxicology and Neurology and scientific director of the VCU Alcohol Research Center, Virginia Commonwealth University, Richmond, Virginia.

**Keywords:** Alcohol use disorder, alcohol research, translational research, treatment research, basic science, clinical science, applied science, systems biology, genetic factors

## Abstract

Alcohol use disorder (AUD) and its sequelae impose a major burden on the public health of the United States, and adequate long-term control of this disorder has not been achieved. Molecular and behavioral basic science research findings are providing the groundwork for understanding the mechanisms underlying AUD and have identified multiple candidate targets for ongoing clinical trials. However, the translation of basic research or clinical findings into improved therapeutic approaches for AUD must become more efficient. Translational research is a multistage process of streamlining the movement of basic biomedical research findings into clinical research and then to the clinical target populations. This process demands efficient bidirectional communication across basic, applied, and clinical science as well as with clinical practitioners. Ongoing work suggests rapid progress is being made with an evolving translational framework within the alcohol research field. This is helped by multiple interdisciplinary collaborative research structures that have been developed to advance translational work on AUD. Moreover, the integration of systems biology approaches with collaborative clinical studies may yield novel insights for future translational success. Finally, appreciation of genetic variation in pharmacological or behavioral treatment responses and optimal communication from bench to bedside and back may strengthen the success of translational research applications to AUD.

More than 20 years ago, Daniel Koshland compared basic with applied research, stating: “Basic research is the type that is not always practical but often leads to great discoveries. Applied research refines these discoveries into useful products” (Koshland 1993). This statement implies that basic science does not have a direct impact on human health and disease or patient outcome but offers the tools that eventually lead to advances in understanding and treating disease states. Application of basic-science knowledge leads to better clinical practices through drug development, improved screening techniques, and better diagnostic tests, to name but a few examples. In the alcohol research field, basic-research discoveries have led to the identification of four of the five medications currently approved for treatment of alcohol use disorder (AUD) in Europe or the United States.

Basic and clinical research have both been clearly defined over the years, but since Koshland’s statement in 1993, a third domain of translational research has been established that offers a bridge between basic research and clinical applications (see figure 1 and table 1). Paraphrasing National Institutes of Health (NIH) Roadmap definitions, “Translation is the process of turning observations in the laboratory and clinic into interventions that improve the health of individuals and the public—from diagnostics and therapeutics to medical procedures and behavioral changes” (http://www.ncats.nih.gov/about/about.html). Under such a definition, basic scientific inquiry might result from basic research, applied research, or clinical research designs. For example, studies on dopamine receptor regulation in animal models, on the effects of ethanol on dopamine receptor gene expression in animal models, or on correlations of dopamine receptor function with the course of AUD using imaging studies in patients could be interpreted as basic, applied, and clinical research, respectively. However, studies assessing whether a drug or behavioral intervention that modulates dopamine receptor function in animals could alter ethanol consumption or toxicity in humans would clearly be an example of translational research.

NIH has placed great emphasis on translational research over the last 10 years, offering strong encouragement via the NIH Roadmap effort and, more recently, the NIH Common Fund. The funding of the Clinical and Translational Science Awards (CTSA) consortium (www.ctsacentral.org) in 2006 and the National Center for Advancing Translational Sciences (NCATS) (www.ncats.nih.gov/index.html) in 2011 are two major manifestations of the increased focus on translating basic-science discoveries into advances in human health. As part of this process, the concept of “bench to bedside” has been formalized and extended. As outlined in table 1, translational research can be described as five phases (T0–T4) that not only encompass traditional clinical trials or clinical research but also include broader areas studying the effective implementation of prevention or treatment advances to patient populations in the community.

The genesis of such a focus on translational research is self-evident. The development of many accomplishments of modern medicine clearly derives from serendipitous or hypothesis-driven observations of basic research. Three of the four medications approved by the U.S. Food and Drug Administration (FDA) for treatment of AUD (i.e., naltrexone, acamprosate, and extended-release naltrexone [Vivitrol®]), as well as an opioid antagonist (i.e., nalmephene) that recently received European market approval for treatment of AUD, were direct products of the translation of basic-science studies (Franck and Jayaram-Lindstrom 2013; Muller et al. 2014). However, complex and extremely common diseases that are currently without fully successful treatment, such as Alzheimer’s disease, cancer, or addictions, have not yielded easily to efforts to translate such basic-research findings into actual new treatments for patients, despite enormous bodies of basic knowledge and what to the lay public seem to be endless NIH expenditures. Success for the existing AUD treatments is only moderate and likely limited by poor compliance, toxicity, individual variation, and possibly lack of full efficacy. Typical hypothesis-driven basic research, despite increasingly complex data derivations and analytical tools, has not produced breakthrough discoveries for complex disease states, comparable to antibiotics or the polio vaccine. Progress has been made, but at a more iterative and deliberate pace than perhaps needed and desired by a public becoming accustomed to instantaneous information access. In concrete terms, the flattening of the NIH budget is both a fiscal and political reality. Thus, emphasis is now being placed on narrowing the gap between basic discovery and clinical application, with the hope that promising advances in knowledge might be applied to human health more rapidly (Woolf 2008). This article discusses some aspects of the process and promise for applying translational approaches in research on AUD and its treatment.

## The Pipeline From Basic Research to Clinical Utilization

**Figure 1:**
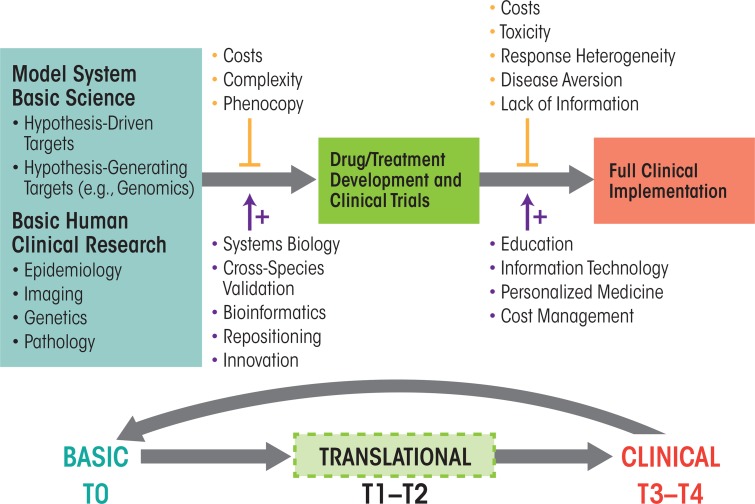
Translational research pipeline. Diagram portraying the information and discovery flow from basic research (left) via translational research (middle) to final clinical application (right). Vertical lines and arrows indicate negative (upper) and supportive (lower) factors modulating the translational pipeline. This process can be thought of as occurring in five stages, from basic research (T0) and translational research (T1 and T2) to clinical research (T3 and T4).

Translational research, as a whole, aims to bridge the gap between basic research, applied clinical research, and clinical practice (figure 1). As mentioned previously, the NIH divides translational research into five separate phases (i.e., stages T0–T4) (table 1 and figure 1), with T0 representing the basic research phase. The first part in the translation process is the application of discoveries from the basic laboratory and pre-clinical studies to the development of clinical trials in humans (i.e., stages T1 and T2). The second part (i.e., stages T3 and T4) involves the translation of clinical-trial research into supporting the best medical practices in the community (Sung et al. 2003). However, for translational research to truly achieve the end goal of better medicine and/or treatments for the public, it must have a two-way relationship with basic science. Thus, on the one hand, the translation of basic knowledge into novel treatments and understanding of disease requires clinical trials that ultimately can generate the information required to transfer discoveries to the public health. On the other hand, clinical observations on disease manifestation and treatment results must in turn be communicated effectively to bench scientists, thereby providing an impetus for further basic investigations of human health and disease. This bidirectionality underlies the concept of bringing the “lab bench to the bedside and back to the bench.” Seen in this fashion, translational research should actually increase the need for funding of both basic science and clinical investigations (Fang and Casadevall 2010).

### Blurring Boundaries Between and Among Basic, Translational, and Clinical Research

Before Koshland’s 1993 statement cited above, basic research historically was limited to academic institutions, offering scientists the freedom to investigate areas of interest, regardless of the likelihood for any future contribution to human health. The discoveries obtained from this basic research led to applied and clinical research, conducted largely by private industry, which generated practical endpoints such as medicines and other approaches or technologies for the treatment of human disease. Today, however, this practice of restricting basic research to academic environments and conducting applied/clinical research in industry settings has changed. This can be observed in the rise of technology transfer offices in major academic institutions across the Nation. This development results at least in part from the introduction of the Bayh–Dole Act in 1980, which allowed universities to patent discoveries made using Federal funds. This avenue allowed scientists to patent knowledge and retain intellectual property rights over discoveries in basic-research laboratories.

The narrowing of the gap between basic and applied/clinical research has created a natural bridge to translational research because of the increased communication occurring between the basic and clinical research arenas. Basic scientists have an intellectual investment in their discoveries, and with the increased availability of technology transfer, they now have an avenue to follow their work from their own bench out into the public by working more closely with clinicians and/or drug-development or medical-technology industries.

However, progress rarely comes without challenges, and this drive to translate basic-science discoveries into immediate practical end points has raised concerns among many prominent scientists who voice their worries that support for basic-science research may potentially decay. Indeed, there is evidence that the push toward translational research has changed the patterns of academic grant submission and funding. In the budget proposal announced by President Obama in April 2013, funding for applied rather than basic research accounted for the majority of the increases seen in the Federal science budget (Hand et al. 2013). This trend further is compounded by the NIH-wide flat budget over the last 10 years, with a net decrease in investigator-initiated R01-type grant applications. R01-equivalent funding success rates now hover at or below 10 percent for most institutes (Fang and Casadevall 2010). Since the lines between basic and applied/clinical science have become blurred, there is concern that NIH grant reviews for basic research increasingly may become weighted based on proposed translational payoffs rather than on the quality of the basic-science aims. However, without basic discoveries, there will be nothing to “translate.” There is thus a crucial need for a proper balance between basic and translational research to adequately feed the scientific pipeline for advancing public health.

## Translating Alcohol Research

AUD is a global health concern. Alcohol can produce a wide spectrum of adverse behavioral and end-organ effects, usually resulting from severe binge drinking or chronic abusive consumption. An estimated 18 million Americans meet the diagnostic criteria for an AUD (http://pubs.niaaa.nih.gov/publications/AlcoholFacts&Stats/AlcoholFacts&Stats.htm). It has become quite clear that ethanol targets specific molecules in the brain and acts, in part, like many other drugs of abuse on reward pathways in the brain. However, ethanol is unique in that it has multiple specific targets for low-affinity interactions, perhaps necessitating different therapeutic approaches to treatment of AUD than for other drug use disorders. Progress in basic science in recent decades has fostered an exciting array of discoveries on the neurobiology of ethanol and AUD. Experiments involving sophisticated animal behavioral models, signal transduction, electrophysiology, neuroimaging, proteomics, gene targeting, human and model-organism genetics, and genomics have all generated data to help researchers and clinicians better understand the molecular mechanisms of ethanol’s actions and the development of AUD (Koob and Volkow 2010; Spanagel 2009).

Despite this progress, at present in the United States only the same four FDA-approved drugs (i.e., disulfiram, naltrexone, acamprosate, and extended-release naltrexone) that existed nearly a decade ago are currently available for the treatment of AUD. Furthermore, three of the five medications approved for treatment of AUD in the United States or Europe (i.e., naltrexone, nalmefene, and extended-release naltrexone) are members of the same class of drugs (i.e., opioid antagonists). These medications represent considerable accomplishments in combating a drug with such diverse sites of action, but there is conflicting evidence regarding their overall impact on managing and reducing the societal cost of AUD (Mann et al. 2013; Mark et al. 2010).

As shown in figure 1, multiple factors complicate the translation of basic research into accepted clinical practice (upper vertical arrows); however, potential solutions to these complications also are available (lower vertical arrows). With regard to ethanol and AUD, problems for translational progress include the presence of multiple sites of ethanol action (which make the research more complex and also increase associated costs), genetic variance in response to therapeutics (i.e., pharmacogenetics); variants of AUD with differing underlying mechanisms, but similar clinical signs (i.e., phenocopy); stigma of alcoholism that prevents people with AUD from seeking treatment; medication toxicity; competing strategies amongst clinicians endorsing psychosocial and/or biological therapies; and uncertainty as to which animal models have greatest predictive validity for therapeutic responses in humans. In light of these complications, how might an increased focus on translational research speed the development of new therapies for AUD or improve the use of current treatments? Several aspects of translational research likely will be crucial in advancing the identification of novel, effective treatment approaches and their implementation on a large scale, including progress in the identification of novel targets for treatments, efforts to advance early translational studies, and approaches to overcome current roadblocks to translational research efforts.

### Improved Target Identification

Identifying targets for therapeutic development has been particularly complex with AUD, most likely because ethanol has multiple low-affinity (albeit specific) molecular sites of action and because of difficulties in identifying animal behavioral models with adequate consilience for AUD (Barkley-Levenson and Crabbe 2012). Recent human genetic studies also suggest that there likely are large numbers of genes modulating risk and/or severity of AUD, each with a relatively small effect on the disease (Edenberg and Foroud 2013). Despite the sizeable impediments posed by target identification for AUD, there has been a remarkable explosion of early-phase clinical trials on a large variety of potential new pharmacotherapeutic and behavioral approaches to AUD treatment (table 2) (see Muller et al. 2014; Spanagel 2009). Studies have functionally linked neurotransmitters such as γ-aminobutyric acid (GABA), glutamate, dopamine, serotonin, acetylcholine, neuropeptide Y, or corticotropin-releasing hormone (CRH) and their respective receptor systems to ethanol responses and/or AUD at the basic-science, preclinical, and clinical-research levels. Additionally, reports from patent databases and smaller clinical studies suggest that other novel pharmacotherapeutic strategies for treatment of AUD may be on the horizon (Muller et al. 2014; Rezvani et al. 2012).

In addition to the productive and active studies mentioned above, rapid progress in high-throughput methodologies, such as DNA micro-arrays, next-generation DNA sequencing, and genome-wide association studies (GWAS), have produced a wealth of potential new targets for translational study in AUD (Farris and Miles 2012; Zhao et al. 2012). The difficulty with such approaches, however, has been deciding how a promising target gene can indeed be identified amongst such large datasets. Some investigators thus have advocated the use of gene networks or pathways as more biologically relevant targets for therapeutic intervention, rather than individual genes within a network (Farris and Miles 2012, 2013; Wolen et al. 2012). Such an approach might, for example, identify a signaling mechanism that regulates a gene network involved in the formation of abusive ethanol-consumption behaviors. Targeting an entire network might obviate the need for laborious prioritizing or validation of individual genes for drug development, thus accelerating the translational pipeline and lending insight into biological functions associated with the drug development.

Other current methodologies lend themselves to rapid validation of genes or networks in behavioral responses to ethanol in animal models. The combination of genomic or proteomic data and genetic studies in animal models, humans, or across multiple species has led to the identification of experimentally tractable novel genes or gene networks with potential for future translational study (Arlinde et al. 2004; Han et al. 2013; Kerns et al. 2005; Zhao et al. 2012). Proposed central “hub” genes regulating target networks or other network regulatory mechanisms for ethanol behaviors can rapidly be validated in animal models through the use of viral vector gene targeting (Bhandari et al. 2012) or recent developments for rapid genetic targeting of specific genes (Gaj et al. 2013). Combining behavioral approaches, such as operant self-administration or other ethanol-seeking and/or alcohol-consumption analyses, that have been validated with FDA-approved pharmacotherapies for AUD (see below) with these gene-targeting approaches may allow progress to translational studies on a tractable number of high-priority targets.

An important role in these new strategies may fall to ambitiously large consortia formed to integrate animal-model and human behavioral, clinical, genetic, genomic, and neuroimaging results, with the goal of identifying and prioritizing key gene networks or signaling systems for future translational studies on AUD (Spanagel et al. 2013). These include efforts spearheaded by the National Institute on Alcohol Abuse and Alcoholism (NIAAA), such as alcohol center grants, Project MATCH, the National Consortium on Alcohol and Neuro-development in Adolescence (NCANDA), the Collaborative Study on the Genetics of Alcoholism (COGA), and the Integrative Neuroscience Initiative on Alcoholism (INIA), as well as the European IMAGEN consortium. Such collaborative studies not only promise to produce vital insight into the basic neurobiological mechanisms underlying the actions of ethanol but also to accelerate translational studies on new treatments for AUD.

### Advancing Early Translational Studies

Although none of the potential therapeutic agents listed in table 2 have yet obtained FDA approval for treatment of AUD, the spectrum of compounds and methods for their study suggests important lessons for translational research on alcoholism. Many of these agents have been identified as candidates through use of multiple basic-science approaches, but with the important inclusion of critical animal models (e.g., two-bottle self-administration, operant self-administration and reinstatement, or alcohol deprivation–induced seeking) that already have been shown to have predictive validation with FDA-approved medications for AUD treatment (Samson 1986). Thus, both naltrexone and acamprosate alter ethanol consumption, deprivation drinking, operant self-administration, or reinstatement of operant responding in animal models (Bachteler et al. 2005; Bienkowski et al. 1999).

Interestingly, many of the drug trials listed in table 2 have employed agents already approved by the FDA for other uses. Such repositioning or off-label use approaches can greatly accelerate translational research. Examples of such agents include topiramate and gabapentin, both of which are FDA-approved anti-seizure drugs. Varenicline, a nicotinic partial agonist approved for treatment of nicotine dependence, has shown promise in some randomized, placebo-controlled trials for treatment of AUD or concurrent nicotine and alcohol dependence (Litten et al. 2013; Mitchell et al. 2012). Major databases of existing clinical trials as well as of drug structure and function (e.g. www.clinical-trials.gov; http://toxnet.nlm.nih.gov; http://druginfo.nlm.nih.gov/drugportal/; and http://www.ncbi.nlm.nih.gov/pcsubstance) also can potentially accelerate preclinical animal studies by consolidating available information, sometimes obviating the need for extensive de novo drug development.

One factor possibly limiting how investigators can evaluate new treatments in clinical trials on AUD is the incidence of false-negative results—that is, studies that as a whole show no statistically significant positive clinical response (e.g., decrease in drinking behavior) even though the desired positive effect of the treatment may occur at the molecular level and be observable in subgroups of subjects. Such false-negative results may be caused by limited clinical assessment measures, underpowered studies, pharmacogenetic factors (e.g., people with a certain genotype do not exhibit the expected response to the treatment), or phenotypic variability. Genetic analyses and neuroimaging studies such as magnetic resonance imaging (MRI), functional MRI, diffusion tensor imaging, and positron emission tomography (PET) can address some of these limitations. Consequently, these approaches have become powerful additional tools that are used not only for human (and animal) basic clinical studies on AUD, but also for evaluating clinical responses to pharmacological agents (Spanagel et al. 2013). In particular, functional neuroimaging has become a widely used translational tool to both identify mechanisms of brain dysfunction in AUD and evaluate potential avenues of intervention (Chanraud et al. 2011; Muller-Oehring et al. 2014; Sullivan et al. 2010). For example, Hirvonen and colleagues (2013) have used PET imaging to demonstrate that alcoholics have profound deficits in the levels of cannabinoid receptor 1 (CB1) in the cortex, and particularly in the frontal cortex, supporting further efforts to pharmacologically manipulate cannabinoid signaling in people with AUD. Further, Schacht and colleagues (2014) demonstrated in a pilot study that varenicline did not reduce the number of heavy-drinking days, but did decrease craving in response to alcohol cues both as reported subjectively and as determined by neuroimaging studies of brain activation in the orbito-frontal cortex. These neuroimaging findings confirmed that varenicline can elicit a clinical response as well as identified regional signaling events in the brain that potentially can be used in further therapeutic development efforts. Finally, a recent preliminary report from the European PREDICT study suggests that genotyping and fMRI studies can identify subgroups of naltrexone responders, thereby potentially improving the yield of clinical trials on treatment of AUD (Mann 2014).

### Overcoming Roadblocks at the Level of Clinical Practice

Some of the major issues regarding implementation of AUD treatment advances in routine clinical care regard a lack of connection between clinical research and the intended population that such research ultimately is targeting (Beckett et al. 2011). A recent study analyzing Veterans Administration records showed that only 3 percent of veterans with an AUD diagnosis were receiving treatment (Harris et al. 2012), even though data from some studies have demonstrated significant overall benefits from FDA-approved AUD treatments in terms of lowering healthcare utilization costs (Mark et al. 2010). This discrepancy between treatment benefit and utilization could be due to a variety of factors, including costs, compliance, medication toxicity, clinician unfamiliarity with available medications or ongoing trials, or the perceived minimal therapeutic response to existing drugs or behavioral strategies for AUD. Therefore, improved treatment for AUD requires not only increased identification of therapeutic targets and effective therapeutic strategies, but also improved communication of existing treatment options and their effectiveness.

Other important factors potentially influencing both the success of later-phase clinical trials and the ultimate utilization of therapies for AUD are the individual variations in treatment responses (which result from pharmacogenetic factors) and the heterogeneity in the diagnosis of AUD (phenocopy). As mentioned previously, multiple ongoing studies have used neuroimaging or genetic analyses to determine outcomes in clinical trials of AUD treatments. Advances in genetic information and technology resulting from intense basic-science research over the last two decades have created the molecular tools for assessing genetic variability in treatment responses. This use of personalized medicine has been demonstrated in recent pharmacogenetic or neuroimaging predictive studies on naltrexone and other pharmacotherapies for AUD (Kranzler and McKay 2012; Mann 2014). Studies demonstrating the effect of variations in individual nucleotides (i.e., single-nucleotide polymorphisms) within the mu-opioid receptor gene (*OPRM1*) on alcohol craving and consumption, as well as on patient responses to naltrexone therapy, offer evidence that genetic factors influence both the risk of AUD and treatment of the disease. Such personalized-medicine approaches likely will be an important component in enhancing the success of future pharmacotherapeutic and possibly behavioral approaches to AUD treatment.

However, such success could be limited by lack of interaction between basic and clinical scientists on the one hand and practicing clinicians on the other. Translational research across stages T3–T4 is a key component to addressing this issue. Clinicians must be made aware of treatments and of pharmacogenetic factors affecting outcome, patients must agree to genetic testing, and this testing must be available to patients. Information about treatment success combined with genetic screenings of patients, in turn, can further advise basic scientists on the neurobiology and genetic components of AUD. Efforts such as the German SysMedAlcoholism consortium represent such a comprehensive approach where genetic, neuroimaging, and clinical results are integrated and analyzed for cross-discipline communication (Spanagel et al. 2013).

## Summary

Translational research is the process of streamlining the movement of findings obtained in basic biomedical research to clinical research and then out into the communities to the patients who are supposed to benefit from such research. This is a complex multistage process that demands effective communication across basic, applied, and clinical sciences as well as with clinical practitioners. To date, researchers face considerable difficulties in the application of such translational-research approaches to the treatment of AUD. However, with multiple existing collaborative frameworks and other ongoing work, there likely will be rapid progress within an evolving translational framework. In particular, molecular, behavioral, and neuroimaging studies in basic or clinical-science research are laying the groundwork for understanding the mechanisms of AUD and alcohol’s end-organ toxicity.

Existing evidence suggests that numerous genetic, clinical, and social factors influence the use and effectiveness of the five existing medications currently approved for treatment of AUD in the United States and Europe. Although multiple potential new targets for therapeutic development already have been identified, modern genetic and genomic studies suggest a more complex molecular framework underlying AUD. Systems-biology approaches combining the identification of gene networks (and their regulatory components) that modulate ethanol consumption/seeking behaviors in validated animal models with human studies on genetic variation in AUD phenotypes or treatment responses may enhance translational speed and success. Optimal communication from the bench to bedside and back may ultimately ensure the success of translational research applications to AUD.

## Figures and Tables

**Table 1:** Definitions for Translational Research Stages From T0 to T4

**Stage**	**Description**
T0: Basic Scientific Discovery	Preclinical or “bench” research directed at mechanisms and presentations of human disease
T1: Translation to Humans	Testing basic science discoveries for clinical effect and/or applicability
T2: Translation to Patients	Testing new interventions in human subjects under controlled environments
T3: Translation to Practice	Research on the application of new interventions or therapies in general practice
T4: Translation to Population	Investigations of factors and/or interventions that influence the health of populations

NOTE: Adapted from “Enhancing the Clinical and Translational Science Awards Program.” Available at: http://www.ncats.nih.gov/files/report-ctsa-rfi.pdf.

**Table 2:** Examples of Agents in Clinical Trials for Treatment of Alcohol Use Disorder (AUD) Since 2009*

**Drug**	**Class/Action**
ABT–436	Vasopressin V1B receptor antagonist
Aprepitant	Neurokinin 1 receptor antagonist
Baclofen	GABA-B receptor agonist
Buproprion	Nicotine receptor partial agonist
Doxazocin	Alpha 1 adrenergic antagonist
Dutasteride	5a reductase inhibitor
Gabapentin	Antiseizure, multiple sites of action
Ghrelin	Neuropeptide
GSK561679	CRH1 receptor antagonist
Ivermectin	P2X4 receptor antagonist
Memantine	NMDA receptor antagonist
Mifepristone	Glucocorticoid receptor antagonist
Mirtazapine	Tetracyclic antidepressant (mixed actions)
Ondansetron	5HT3 receptor antagonist
Pioglitazone	PPAR agonist
Topiramate	Antiseizure, multiple sites of action
Varenicline	Nicotinic receptor partial agonist

NOTE:

* Not including behavioral treatments, FDA- or non–FDA-approved agents, or modifications of medications with existing FDA approval for treatment of AUD.

SOURCE: www.clinicaltrials.gov.
